# Proceedings from the CIH^LMU^ Symposium 2020 on “eHealth: Trends and innovations”

**DOI:** 10.1186/s12919-020-00202-3

**Published:** 2020-12-07

**Authors:** Ivan Noreña, Nairuti Shah, Jackson Ndenkeh, Cecilia Hernandez, Nadia Sitoe, Abdou Sillah, Anna Shin, Wai Wai Han, Yoga Devaera, Maureen Mosoba, Given Moonga, Tereza Hendl, Alina Wernick, Vincent Micheal Kiberu, Melissa Menke, Jessica Michelle Guggenbuehl Noller, Michael Pritsch

**Affiliations:** 1 CIHLMU Center for International Health, University Hospital, LMU Munich, Munich, Germany; 2 Division of Infectious Diseases and Tropical Medicine, University Hospital, LMU Munich, Munich, Germany; 3 Instituto Nacional de Saúde, Maputo, Mozambique; 4 Medical Research Council Unit the Gambia, London School of Hygiene and Tropical Medicine, Fajara, The Gambia; 5 Department of Medical Research, Ministry of Health and Sports, Naypyidaw, Myanmar; 6 Department of Epidemiology and Biostatistics, University of Zambia, Lusaka, Zambia; 7 Institute of Ethics, History, and Theory of Medicine LMU, Munich, Germany; 8 Alexander von Humboldt Institute for Internet and Society, Berlin, Germany; 9 Makerere University School of Public Health, Kampala, Uganda; 10 Access Afya, Nairobi, Kenya

**Keywords:** eHealth, Public health, Implementation science, Technology transfer, Universal health care

## Abstract

Electronic Health (eHealth) is the use of information and communication technologies for health and plays a significant role in improving public health. The rapid expansion and development of eHealth initiatives allow researchers and healthcare providers to connect more effectively with patients. The aim of the CIH^LMU^ Symposium 2020 was to discuss the current challenges facing the field, opportunities in eHealth implementation, to share the experiences from different healthcare systems, and to discuss future trends addressing the use of digital platforms in health. The symposium on eHealth explored how the health and technology sector must increase efforts to reduce the obstacles facing public and private investment, the efficacy in preventing diseases and improving patient quality of life, and the ethical and legal frameworks that influence the proper development of the different platforms and initiatives related to the field. This symposium furthered the sharing of knowledge, networking, and patient/user and practitioner experiences in low- and middle-income countries (LMIC) in both public and private sectors.

## Background

eHealth is the use of information and communication technologies (ICT) for health. The World Health Organization (WHO) has acknowledged the potential of digital technologies playing a significant role in improving public health [1]. The resolution urges the member states to prioritize the development and greater use of digital technologies in health as one of the means to promote universal health coverage and advancing the sustainable development goals (SDGs) [2]. eHealth was created to address the gaps existing between patients and healthcare professionals. The expansion of eHealth solutions is associated with the increased demand for flexible, comprehensive, as well as cost-effective care models, and as it grows, puts together a very exhaustive set of knowledge and multidisciplinary efforts. However, there are still several regions in which legal or ethical framework to supervise eHealth development are lacking.

## Summary of presentations

### Concepts of public health informatics and DHIS2

**Michael Pritsch, MD.**

Division of Infectious Diseases and Tropical Medicine, University Hospital, LMU Munich, Germany. Clinical scientist and coordinator of the working group Global Health Informatics.

**Jessica Michelle Guggenbuehl Noller, MSc.**

Division of Infectious Diseases and Tropical Medicine, University Hospital, LMU Munich, Germany. Project manager and researcher.

## Overview

There has been an exponential increase in global creation of data, including data concerning health in recent years. However, converting available data into useful health information and indicators can prove difficult; thus, it is seldom achieved in a comprehensive manner. Major challenges in this field are data overload and poor data quality. Both can spiral into a so called “vicious data cycle”: Data overload impedes appropriate data use, which can urge clinicians and researchers to seek out for even more data. As a result, more data is collected, leading to even more data overload. Poor data quality leads to infrequent data use or data neglect, which enhances the deterioration of data quality. The concept of “open data” increases information transparency and accountability, and thus can potentially benefit society. Free and open-source software like the District Health Information Software 2 (DHIS2) can help with reporting, analysis, and dissemination of data with potential use cases on a local, national, and global level [3]. For example, the use of DHIS2 for antimicrobial resistance (AMR) surveillance and as a decision support tool for medical staff was presented as a beneficial approach to improve antimicrobial stewardship interventions [4].

## Discussion

- Funding is a relevant barrier to the implementation of these open digital platforms into public health programs.

- Continuous data use even of incomplete or lower quality data, can lead to improved data quality.

- Implementation of inappropriate tools for data collection can increase the workload in healthcare professionals triggering burnout. Thus, tools need to be evaluated carefully not to increase strain on medical staff, but rather lower workload.

### Beyond the hype: opportunities and challenges in mHealth

**Tereza Hendl, Ph.D.**

Institute of Ethics, History, and Theory of Medicine, LMU Munich, Germany. Researcher and Project Co-lead on the project “META – mHealth: Ethical, legal and societal aspects in the technological age.”

## Overview

Mobile health (mHealth) is a rapidly developing field, involving a range of devices, including wearables, sensors, or mobile software, such as *apps* [2]. Advocates of mHealth have argued that these technologies can transform the healthcare system by facilitating adequate healthcare access to more people, democratizing healthcare —whereby healthcare is made accessible to everyone—, and empower patients and users. However, this approach faces many potential challenges calling all lack of accessibility to many contemporary healthcare facets that can lead to a digital divide, where patients and users are ostracized by their available technological means. mHealth technologies are being introduced in a world with a persistent digital divide as well as many structural inequalities (e.g. on the grounds of regional location, class, gender or ethnicity). These power imbalances not only limit individual access to the platforms but also opportunities for one’s participation in the design of these technologies, which is reflected in their content. In this regard, some have cautioned that mHealth can exacerbate some inequalities and vulnerabilities in the field of healthcare. Some of the novel aspects of mHealth technologies —such as their work with digital data— generate concerns as power asymmetries can translate into algorithmic bias. Further concerns include data privacy and security, changing roles of health professionals, the undermining of empathy in telemedicine as well as the erosion of trust in the doctor-patient relationship. The chances and risks associated with mHealth as well as the individual, social, and global impact of these technologies need to be continuously evaluated, and more empirical research investigating the efficacy and health benefits of particular mHealth technologies is warranted.

## Discussion

- The potential scale (e.g. local and global) and social implications of these platforms are significant, hence, there is a need for a robust discussion about their ethical, social and legal aspects in the context of their potential integration into healthcare systems.

- The viability of “*app* prescription” as a routine clinical intervention is still debatable and should be reserved for the platforms with shown safety and efficacy.

- Defining who owns mHealth data and how the data can be handled depends on regulatory frameworks, such as the general data protection regulation (GDPR). However, the international and global character of mHealth platforms poses significant legal challenges that require further debate.

### Regulatory aspects of eHealth: could soft law bring more cohesion?

**Alina Wernick, LL.M.**

Associated researcher, Humboldt Institute for Internet and Society, Research program “Data, Actors, Infrastructures. The governance of data-driven innovation and cybersecurity.” Berlin, Germany.

## Overview

Presently, the regulatory framework for the development and implementation of eHealth initiatives is one of the more challenging topics in the field. The relevant legal landscape is complex because it involves numerous fields of law related to the health and technology sectors. Relevant legislation varies from country to country, often reflecting differences between national healthcare systems. As a result, there is no universal legal framework for eHealth. Global trends usually lead to increasing regulations, and national laws addressing eHealth have been passed at an accelerating pace. However, we find notable differences among regions. In 2016, the WHO report on eHealth showed a predominant diffusion of eHealth on high-income countries, in parallel with a higher rate of eHealth regulation developments compared to other regions [2]. The digitalization of the health sector creates situations, where existing legislation may be difficult to apply to eHealth, leave it unregulated or enables ethically grey areas to emerge.

On the international level, fundamental rights create, at least indirectly, safeguards and certain congruence in the national laws governing eHealth. However, given the scarcity of other internationally binding instruments in the context of eHealth, the main tool to influence regulation of eHealth on the international level is policy cooperation and legally non-binding soft law, such as SDGs and the WHO’s resolutions and guidelines.

## Discussion

- The national/regional background in health and technology laws strongly determines the current robustness or fragility in the regulation system. Strategies to strengthen the regulatory framework include evidence-based assessment of technologies and efforts to find a balance between promoting safety, security, and innovation in eHealth with consideration of the local health care setting.

- The advent of novel, often convergent technologies reveal weaknesses in the regulatory systems, demanding responses to realign the former legal framework or the enactment of entirely new laws.

- Geopolitical interests and economic trends surrounding technologies such as artificial intelligence has an impact on eHealth regulation. A holistic view is crucial for identifying the appropriate scope and level (regional/national/supranational) of legislation addressing eHealth.

### From models to real-world scenarios: eHealth implementation in low- and middle-income countries

**Vincent Micheal Kiberu, Ph.D.**

Makerere University School of Public Health, Kampala, Uganda. Researcher at the Health Informatics Research Group.

## Overview

Several challenges play a role in setting back the successful implementation of eHealth initiatives in LMIC. All lack of funding in both private and public healthcare is one of the most critical challenges for the implementation of eHealth in these regions, followed by sustainability, technology infrastructure, and connectivity. The implementation of DHIS2 platform in Uganda at a national level is an excellent example of the strengthening of the reporting network on a nation-wide scale. This tool addressed difficulties related to poor data quality from a public health perspective. After several training workshops for users, they achieved a significant improvement in health information reporting. Nevertheless, this initiative also exhibited the different barriers in the implementation process as limited access to computers and internet, inadequate technical support and limited worker force [5]. These challenges in the implementation of eHealth are also widely present in LMIC and different multidisciplinary strategies should be planned to manage them in the early phases of development.

## Discussion

In LMIC, eHealth initiatives hatched in the 1990s but its potential gained momentum in the early 2000s, focusing on addressing basic access and quality issues as well as cost inefficiencies in healthcare.

- In LMIC, cooperation of different national institutions aside from the Ministry of health is needed to better succeed in the implementation of these platforms in a national-wide basis and facilitating sustainability.

- Connectivity and proper infrastructure are essential elements for the development of eHealth initiatives; thus, they should always be assured to appropriate sustainability, ensuring continuous operation of eHealth platforms in LMIC**.**

- Ongoing training of professionals from LMIC on eHealth is essential to optimize the workforce required for development, implementation, and sustainability of these initiatives. LMIC universities should increase the offer of academic programs on eHealth related fields to achieve this goal.

### Accessibility and impact on eHealth from Africa

**Melissa Menke.**

Access Afya, Nairobi, Kenya. Founder and CEO.

## Overview

The development of eHealth initiatives in LMIC in many scenarios also depends on the contributions of the private sector. In several very low-resourced settings, access to health services is still a critical problem. Access Afya is a social enterprise that provides affordable, convenient, and effective health access in Kenyan neighborhoods with extreme poverty using eHealth to optimize the functioning of their services, improving the beneficial impact on communities [6]. This company structured a healthcare system with facilities for medical visits, laboratory samples transport/processing, and promotion of health in the community with the use of an eHealth platform that allows integration of all these services. Access Afya also focuses on field programs to extend the reach to schools and factories using mHealth and backpack health programs. On the eHealth side of the initiative, there are digital therapeutic packages that include virtual support (telemedicine), access to medications and testing.

## Discussion

- Funding is relevant to the development and sustainability of this kind of initiative. In the case of Access Afya, funding sources are mixed from donors and investors. Additionally, they charge an affordable fee for services to the community.
- Training and involvement of people from communities in the project are essential to facilitate the integration of services satisfying their needs.
- Different socio-cultural barriers could be challenging for the community when they are looking for healthcare facilities. Therefore, active surveillance using mHealth emerges as a useful strategy that facilitates the follow-up of the population that uses this kind of service.

## Panel discussion

**Michael Pritsch, Jessica Michelle Guggenbuehl Noller, Tereza Hendl, Vincent Micheal Kiberu and Melissa Menke.**

- The cultural background is an important factor influencing the implementation of eHealth platforms. The pre-evaluation of cultural aspects for patients/users and healthcare workers should be emphasized to improve acceptability. Consequently, it is recommended that communities intended to benefit from these platforms be involved in the development of these initiatives since the early phases.
- Now, defining the fundamental elements to establish the threshold between a “good” or “bad” eHealth initiative is quite challenging. Nevertheless, transparency in data handling, evidence-based demonstration of efficacy, and self-evaluation/regulation should be key objectives for developers. These actions could define boundaries and help developers and users in the evaluation as well as identification of beneficial initiatives.
- In the future, good interoperability between different initiatives is necessary. The improvement in regulations could also influence the achievement of this integrative angle. That will also allow the availability of more homogeneous data that could be useful for interpretation.
- Social media are still having a severe lack of data management regulations making it hard to incorporate these platforms to public and global health programs. However, pending optimal use, they could be beneficial in some cases.
- In general, the eHealth implementation process in a nationwide scenario needs to be supported on four pillars: sustainability, adaptability, accessibility, and integrative approach. These factors will allow long-lasting interventions with better incorporation into the healthcare system, reaching more benefits for patients or target populations.

## Final remarks and conclusions

The use of internet technologies and mobile phones is increasing every year worldwide, but more significant increases in LMIC have been observed. Despite that by 2019, just 19% of individuals from resource-limited countries had access to the internet [7], these increasing trends have been taken as an excellent opportunity for developing and implementing eHealth technologies in these regions. Further fueling this premise, the WHO Global Observatory for eHealth in the third global survey report in 2016 exhibited that 80% of the African region countries had at least one type of mHealth program accessing/providing health services [2].

Nevertheless, several challenges are still facing for the implementation and success of eHealth initiatives in LMIC [8–11]. First is to obtain initial and sustainable funding, making necessary collaboration between stakeholders from the public and private sectors [11]. Additionally, based on the best practices in scaling digital health in LMIC proposed by Labrique et al. [10] is still finding it challenging to find user-centered program design enrolling the potential users in the development process: looking for adaptations based on the socio-cultural background crucial for the subsequent acceptance of these platforms. Moreover, in program design, transparency of data is still a challenging issue, providing trustful information with quality (useful) avoiding violating user data privacy.

The human factor is also a critical issue that could affect the sustainability of eHealth initiatives in LMIC regarding frequent lack of a trained workforce, lack of clear roles, and worker burnout [11]. On the other hand, technical factors are often critical limitations concerning poor infrastructure and connectivity limiting technology implementation. Furthermore, many initiatives need better interoperability, primarily with national ministries of health to allow more integrative approaches of these tools to the healthcare system, and even with external systems like communications and energy sectors.

Presently, one of the difficulties that strongly influence many of the previously mentioned barriers is the lack of regulation. Currently, the eHealth legal framework has many unregulated or gray areas that even increase with the advent of entirely novel technologies. The WHO report revealed that policies or legislation to define medical jurisdiction, liability, or reimbursement of eHealth by 2016 was present just in 5% of low- and around 15% of middle-income countries [2]. Collective efforts from local and global authorities in the technology and health sectors to generate policies on eHealth are needed to assess and facilitate cooperation, transparency, and real benefit and impact of these initiatives.

To conclude, many initiatives and stakeholders are investing all kinds of resources to the development of beneficial tools on eHealth, focusing on the improvement of living and health conditions worldwide, including LMIC. While finding a perfect balance and boundaries could be challenging in today’s world considering many gaps in the field, if developers look for transparency, adaptability, accessibility, sustainability, and integration, this could ultimately give valuable healthcare initiatives. For this, the cooperation from different levels and fields is essential to strengthening the eHealth incorporation and efficacy with the conventional health services making closer the way in achieving the projected SDGs.

## Data Availability

NA

## Abbreviations

eHealth: Electronic health
ICT: Information and communication technologies
LMIC: Low- and middle-income countries
WHO: World health Organization
SDGs: Sustainable development goals
DHIS2: District health information software 2
AMR: Antimicrobial resistance
mHealth: Mobile health
GDPR: General data protection regulation
